# Accurate measurement techniques and prediction approaches for the in-situ rock stress

**DOI:** 10.1038/s41598-024-64030-7

**Published:** 2024-06-09

**Authors:** Peng Li, Meifeng Cai, Shengjun Miao, Yuan Li, Liang Sun, Jiangtao Wang, Mostafa Gorjian

**Affiliations:** 1 Key Laboratory of Ministry of Education for Efficient Mining and Safety of Metal Mines, University of Science and Technology Beijing, No.30 Xueyuan Road, Haidian District, Beijing, 100083 China; 2 State Key Laboratory of Coal Resources and Safe Mining, China University of Mining and Technology, Xuzhou, 221116 China; 3 Key Laboratory of Intelligent Bionic Unmanned Systems of Ministry of Education, University of Science and Technology Beijing, Beijing, 100083 China; 4 Geological Engineering, Department of Earth, Ocean and Atmospheric Sciences, University of British Columbia, Vancouver, BC Canada

**Keywords:** Accurate measurements of in-situ stress, Improved overcoring technique, Stress prediction, Embedded GM–BPNN model, Civil engineering, Statistics

## Abstract

The precise calculation and evaluation of the in-situ rock stress tensor is a crucial factor in addressing the major challenges related to subsurface engineering applications and earth science research. To improve the accuracy of in-situ stress measurement and prediction, an improved overcoring technique involving a measurement circuit, temperature compensation, and calculation method is presented for accurately measuring the in-situ rock stress tensor. Furthermore, an embedded grey BP neural network (GM–BPNN) model is established for predicting in-situ rock stress values. The results indicate that the improved overcoring technique has significantly improved the stress measurement accuracy, and a large number of valuable stress data obtained from many mines have proved the testing performance of this technique. Moreover, the mean relative errors of the prediction results of GM(0, 1) for the three principal stresses all reach 6–30%, and the accuracy of the model fails to meet the requirements. The average relative errors of the prediction results of the BPNN model are all less than 10%, and the model accuracy meets the requirements and has sufficient credibility. Compared with the GM and BPNN models, the embedded GM–BPNN model produces the best results, with mean relative errors of 0.0001–4.8338%. The embedded GM–BPNN model fully utilizes the characteristics of grey theory and BP neural network, which require a small sample size, weaken the randomness of the original data, and gradually approach the accuracy of the model, making it particularly suitable for situations with limited stress data.

## Introduction

The precise calculation and evaluation of the in-situ rock stress tensor is a crucial factor in addressing the major challenges related to the design, construction, and operation of subsurface engineering, such as mining, civil, and petroleum projects^1–6^. Meanwhile, accurate in-situ stress data is also indispensable for earth science research, such as plate driving mechanism, tectonic evolution, earthquake mechanism and prediction, and fault activity^7–14^. It is well known that the genesis of in-situ stress is extremely complicated due to the long-term countless tectonic movements experienced by the earth as well as various geological structures with different scales and directions widely developed within the earth, and the crustal stress field exhibits remarkable temporal and spatial characteristics. As a consequence, the complexity and variability of the crustal stress state are caused, and its performance varies in regions with different geological tectonical backgrounds.

Compared with other rock mass properties, rock stress is a difficult quantity to measure. As Leeman^15^ pointed out, “It is impossible to measure stress directly since, in fact, it is a fictitious quantity. It is only possible to deduce the stresses in a solid body from the results of measurements using some indirect method”. At present, reliable information about the magnitude and direction of in-situ stress can only be obtained by in-situ measurement technique. Over the past decades, various techniques for determining in-situ stresses, such as hydraulic methods, overcoring methods, jacking methods, strain recovery methods, and borehole breakout methods, have been developed and improved. Generally, all in-situ stress measurement techniques involve disrupting the rock. The response related to the disturbance is measured (in the form of strain, displacement, or hydraulic pressure record) and analyzed by making several assumptions about the constitutive behavior of rocks, and the process of disturbance itself is usually accounted for in the analysis^16^. The identified in-situ stress is a point tensor quantity and the defined stress values have uncertainties associated with site geology and environmental factors (including topography, rock type, geological structures, and anisotropy and heterogeneity of rocks), the accuracy of various measurement methods, and data analysis and interpretation approaches^17^. Hence, understanding and controlling all known sources of error is crucial. According to Amadei and Stephansson^18^, 10–20% scatter in stress magnitude and ± 20° scatter in stress directions have to be accepted when conducting a series of stress measurements in typical rock conditions. Against this background, considerable efforts have been devoted to improving in-situ stress testing equipment and enhancing measurement accuracy worldwide recently.

In general, the measurement of in-situ stress is to determine the undisturbed three-dimensional stress state existing in the stratum, which is usually achieved through point-by-point measurements. To accurately determine the in-situ stress in a certain area, a large number of “point” measurements are necessary. However, due to the limitation of various factors such as manpower, material resources, site, and funds, it is impossible to measure the stress at all points in the target area, but only at a few points, so the measured stress data often has strong randomness, large dispersion, and small sample size. When using traditional probability statistics methods to simulate and fit the stress field of the entire target area and establish a fine stress field distribution model based on the limited measured data, the calculation error will be significant. If the non-linear fitting approach is applied to the limited measured data, the convergence and accuracy of the fitting results cannot be guaranteed. Moreover, although some other popular prediction methods^19–25^ such as multivariate regression, machine learning, deep learning, numerical simulation, neural networks, support vector machines, genetic algorithms, and random forest models have been developed and have emerged as key players in various industries, these methods are mainly devoted to mining the implicit rules of the dataset from a large amount of data, predicting or classifying expected results, and have problems such as too many iterations, slow convergence speed, and is prone to get stuck in local minima. Furthermore, the current research on in-situ stress prediction methods primarily focuses on theoretical models (such as rock physical equivalent model)^26^ and numerical simulation methods (such as boundary displacement adjustment method and displacement function method)^27^, while there are few studies on the prediction of in-situ stress by using new mathematical methods, such as coupling of two single mathematical methods. Thus, to overcome these shortcomings, it is particularly necessary to develop effective and accurate approaches for predicting in-situ stress.

To that end, in the present study, taking the most commonly used overcoring method as an example, some innovative techniques, equipment, and measures developed to improve the accuracy of in-situ stress measurement are systematically introduced. Moreover, the improved overcoring method has been used to measure the in-situ stress in many mining areas in China in recent years, and a large number of valuable and high-quality stress data have been obtained. These measurement practices have proven that the measurement accuracy and reliability of in-situ stress have been significantly improved. In addition, to address the shortcomings of the current stress prediction approaches, an improved grey neural network combination model is proposed based on the grey system theory and neural network principle, and the model algorithm is synchronously optimized. All the equations are fully developed, and the model is put forward in its entirety. Furthermore, taking the compiled measured stress data as sample data, the successful application of this model is also presented, and the predicted results are compared with the in-situ stress value determined by a single grey model and a single neural network model. This combination model only requires a small sample size, can weaken the randomness of the original data, and can be continuously modified to converge to satisfactory accuracy. The achieved results provide a scientific basis and method for greatly improving the accuracy of in-situ stress measurement and prediction.

## Overcoring stress measurements

### Improved overcoring technique for accurate measurement

**Figure 1:**
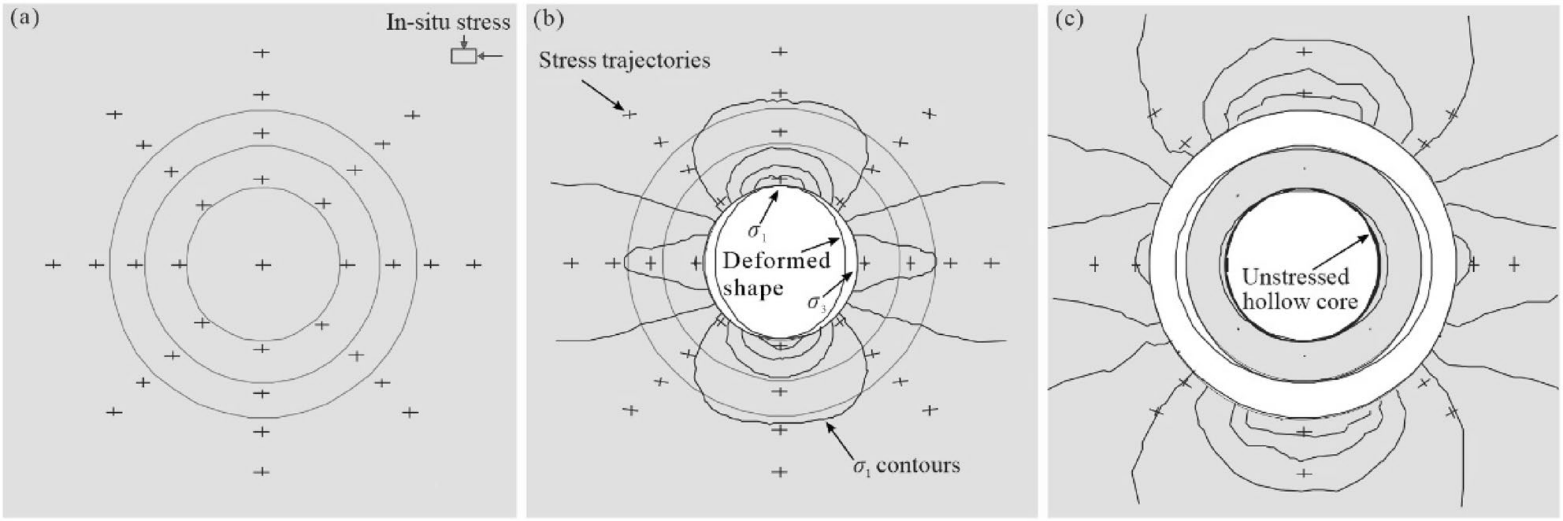
Schematic diagram of the stress state at the vicinity of the borehole at different phases during OC (after Hakala et al.^17^): in-situ state (**a**); after borehole drilling (**b**); and after OC (**c**).

The overcoring (OC) method is an indirect in-situ stress measurement technique that can accurately and quantitatively determine the original rock stress vector. It is currently the only method that can obtain the magnitude and direction of the complete three-dimensional stress from a single OC test^28,29^. The OC method is based on measuring the response strain when the rock core is released from the stress field of the surrounding rock mass, and the original rock stress vector can be estimated by analyzing the measured strains with the knowledge of the elastic properties of rocks^6^. Normally, the induced strains are measured by 12 strain gauges with different orientations before, during, and after the stresses on the borehole wall are released through OC (Fig. 1)^17^. The strain difference is used to back-calculate the stresses acting on the rock cylinder prior to OC assuming continuous, homogeneous, isotropic, and linear-elastic rock behavior. In the early 1950s, Hast^30^ first applied the OC method to measure in-situ stresses in Scandinavia. After decades of theoretical and technical development, this method has become one of the recommended methods for determining rock stress recommended by the Test Methods Committee of the International Society for Rock Mechanics (ISRM) in 2003^31^. Among the various OC devices developed, the OC technique with a hollow inclusion strain gauge is the most representative one. Since the CSIRO-HI type strain gauge was invented in the 1970s^32^, this technique has been extensively adopted in practice worldwide. According to statistics, among various stress indicators, the in-situ stress data measured by the OC method accounts for approximately 80%.

The nature of rock (discontinuity, heterogeneity, and anisotropy), changes in environmental temperature at measurement points, and the selection method of rock elastic parameters used in stress calculation are the three main factors that affect the accuracy of in-situ stress measurement. The conventional OC method for solving the original rock stress is based on the basic assumption of elastic theory^33^, that is, the rock is homogeneous and isotropic. However, most rock masses have anisotropic properties. Anisotropy leads to the measured stress value being larger than the actual value in some directions and smaller than the actual value in other directions. Thus, the influence of rock anisotropy must be considered when calculating in-situ stress based on the measured strain value of stress relief. For rock masses with simple anisotropic properties such as transverse anisotropy and orthogonal anisotropy, anisotropic theoretical analytical solutions or numerical simulations can be used for the analysis and calculation of measurement results. However, for most rock masses, the combination of anisotropy, discontinuity, and heterogeneity is extremely complex and cannot be described using a simple model. For this purpose, the effect of anisotropy can be preliminarily corrected by using the results of the confining pressure test of the borehole core. In addition, the outstanding advantage of the hollow inclusion strain gauge is that the strain gauge and the borehole wall are bonded together in a considerable area, thus ensuring good bonding quality. Moreover, the cementing agent can also be injected into the cracks and defects in the surrounding rock around the strain gauge to complete the rock mass, making it easier to obtain a complete borehole rock core. Thus, the hollow inclusion strain gauge can be used in moderately discontinuous and uneven rock masses and has good waterproof performance. This has been confirmed in previous stress measurement practices^34^.

Hollow inclusion strain gauges, like many other strain measuring instruments, use resistance strain gauges as measuring elements and convert strain changes into resistance changes and then voltage changes based on the Wheatstone bridge principle for measurement and recording. The resistance strain gauge is quite sensitive to temperature, and its resistance values will change accordingly when the temperature changes, generating corresponding output voltage in the bridge. As a result, the false additional strain values will be calculated. To eliminate this additional temperature strain value, corresponding compensation measures must be taken^35^. For this reason, Cai et al.^36–38^ invented a complete temperature compensation technique, which includes the following points: (1) in the Wheatstone bridge used to measure and record the strain value of each strain gauge, except for the working strain gauge, the three bridge arms are all ultra-low temperature coefficient resistors, and their resistance values basically do not change with temperature, so they will not cause output voltage in the bridge when the temperature changes; (2) monitoring the temperature changes of the strain gauge during the stress relief; (3) after the stress relief is completed, the drilled core containing a hollow inclusion strain gauge is placed in an incubator with adjustable temperature for temperature calibration test to determine the temperature strain rate (i.e., the strain value generated by a temperature change of 1 °C) for each measuring point and strain gauge; and (4) based on the calibrated temperature strain rate and the measured temperature change at the measuring point during the stress relief, the false additional strain value of each strain gauge caused by temperature changes during the stress relief can be calculated, and this additional strain value is removed from the measured total strain value to obtain the true strain of each strain gauge caused by the stress relief. In addition, the wires of the resistance strain gauge can also cause significant additional strain when the temperature changes, so this part of the additional strain should be compensated correctly.

**Figure 2:**
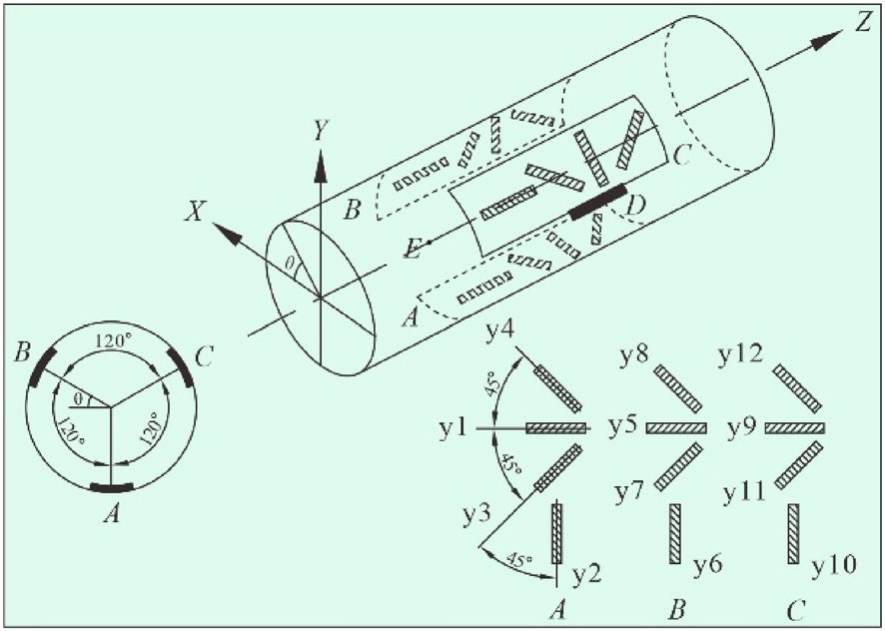
Schematic diagram of the structure of the improved hollow inclusion strain gauge^43^.

To use the complete temperature compensation technique, significant improvements have been made to the structure of the traditional hollow inclusion strain gauge^39,40^, as illustrated in Fig. 2. The related improvements mainly involve embedding a thermistor at the point *D* between two sets of strain gauges for continuous measurement of temperature changes of the strain gauge during OC^41^. Furthermore, at point *E*, two wires that are the same as the strain gauge wires are welded together, and are led out of the cable along with other wires and connected to a bridge arm adjacent to the working strain gauge. As such, the additional strain of the strain gauge wires due to temperature change during stress relief can be offset based on the principle of the Wheatstone bridge. Moreover, the traditional common compensation patch is removed in the improved hollow hollow strain gauge, as this type of compensation patch is not suitable for the temperature compensation of cemented strain gauges. In addition, since the ordinary resistance strain gauge cannot be used for the aforementioned complete temperature compensation technique, a dedicated strain-resistance–voltage conversion device is designed and developed based on the principle of the Wheatstone bridge. This device contains 12 bridges, and each bridge is used for data measurement of a strain gauge. The voltage output from the 12 bridges is automatically recorded by the data collector, which can simultaneously collect the data from 48 channels in one-thousandth of a second, eliminating the influence of the contact resistance of the ordinary resistance strain gauge conversion switches on the measurement results.

In addition, the measurement accuracy of the elastic modulus and Poisson’s ratio, especially the elastic modulus, of rocks, has a great influence on the accuracy of the in-situ stress calculation results, so it is of great significance to improve the measurement accuracy of the elastic parameters to improve the accuracy of the stress measurement results^42,43^. In the existing in-situ stress measurements, it is assumed that the rock is linearly elastic, and the influence of stress levels on the elastic modulus and Poisson’s ratio is ignored when calculating the elastic modulus and Poisson’s ratio of rocks through uniaxial compression tests or biaxial confining pressure tests. In fact, most rock masses are not completely linear elastic, and the elastic modulus of rocks varies with the stress level. Generally, the elastic modulus at a high-stress level is much larger than that at a low-stress level. Hence, calculating low-stress values using elastic modulus values under high-stress levels or calculating high-stress values using elastic modulus values at low-stress levels will cause considerable errors in the calculation results. Meanwhile, for nonlinear rock masses, the loading and unloading paths are different, and the calculation of the elastic modulus from the loading path can also cause certain errors because stress relief is an unloading process. Consequently, to consider the nonlinear elasticity of rocks, the elastic modulus value used must be in accordance with the calculated stress level.

### Theoretical calculation

For the improved OC technique, after the temperature calibration, the final strain values measured by the hollow inclusion strain gauges during the stress relief process can be used to calculate the in-situ stress values according to the following formulas:

1 $$\varepsilon_{\theta } = \frac{1}{E}\left\{ {\left( {\sigma_{x} + \sigma_{y} } \right)K_{1} + 2\left( {1 - \upsilon^{2} } \right)\left[ {\left( {\sigma_{y} - \sigma_{x} } \right)\cos 2\theta - 2\tau_{xy} \sin 2\theta } \right]K_{2} - \upsilon \sigma_{z} K_{4} } \right\}$$

2 $$\varepsilon_{z} = \frac{1}{E}\left[ {\sigma_{z} - \upsilon \left( {\sigma_{x} + \sigma_{y} } \right)} \right]$$

3 $$\gamma_{\theta z} = \frac{4}{E}\left( {1 + \upsilon } \right)\left( {\tau_{yz} \cos \theta - \tau_{zx} \sin \theta } \right)K_{3}$$

4 $$\varepsilon_{ \pm 45^\circ } = \frac{1}{2}\left( {\varepsilon_{\theta } { + }\varepsilon_{z} \pm \gamma_{\theta z} } \right)$$

where *ε*_*θ*_, *ε*_*z*_, *γ*_*θz*_, and *ε*_±45°_ represent the circumferential strain, axial strain, shear strain, and the strain at 45° to the axis, respectively; *σ*_*x*_, *σ*_*y*_, *σ*_*z*_, *τ*_*xy*_, *τ*_*yz*_, and *τ*_*zx*_ are the six components of the original rock stresses; *E* and *υ* are the elastic modulus and Poisson’s ratio, respectively; *θ* is the separation angle between the strain gauge and the X-axis; and *K*_1_, *K*_2_, *K*_3_, and *K*_4_ stand for the four correction coefficients, which are collectively referred to as *K* coefficients.

In the hollow inclusion strain gauge, three groups of strain gauges are embedded in the center of an epoxy cylinder wall instead of directly sticking to the surface of the strain gauge, and the measured strain value is different from that of the strain gauges directly sticking to the borehole wall. The four correction coefficients are used to correct this difference, and the values of the four correction coefficients are related to the elastic modulus and Poisson’s ratio of rocks and hollow inclusion materials, the inner and outer diameters of hollow inclusions, and the radial position of strain gauges in hollow inclusions. Only for different rocks, the values of these correction coefficients can differ by 50%. Therefore, for each OC measuring point in a borehole, the values of the four correction coefficients must be calculated using the formulas provided by Duncan Fama and Pender^44^.

The elastic modulus and Poisson’s ratio of the rock are necessary when calculating the in-situ stress from the strain value measured by the hollow inclusion strain gauge during the OC process. The elastic modulus and Poisson’s ratio of the cored rocks at each measuring point are obtained through confining pressure calibration experiments of the drilled rock cores, which are retrieved from the site after each stress relief test, thus ensuring that the elastic modulus and Poisson’s used for stress calculation truly correspond to the rocks at the measuring points. The specific method and process of the confining pressure calibration experiment can be found in the detailed introduction by Cai et al.^37^. Moreover, considering the influence of colloid deformation parameters and bonding gap, the calculation formula of elastic modulus is revised:

5 $$E = K_{{1}} \left( {\frac{{P_{{\text{c}}} }}{{\varepsilon_{\theta } }}} \right)\frac{{2R^{2} }}{{\left( {R^{2} - r^{2} } \right)}}$$

6 $$\upsilon { = }\frac{{\varepsilon_{\theta } }}{{\varepsilon_{z} }}$$

where *P*_c_ is the confining pressure; and *r* and *R* are the inner and outer radii of the core, respectively. Note that in the previous calculation of elastic modulus, *K*_1_ was assumed to be a fixed constant of 1.12^43^. In some cases, *K*_1_ may be equal to or close to this value, but for many rocks, especially soft rocks, *K*_1_ is not equal to 1.12, and its value is between 0.80 and 1.20. Replacing *K*_1_ with 1.12 will cause a great error in the calculation results of rock elastic modulus, with an error as high as 40%.

In summary, in the OC method, when calculating the rock elastic modulus, the coefficient *K*_1_ is required, and when calculating the coefficient *K*_1_, the rock elastic modulus also needs to be known. Thus, the iterative method is needed to solve. At the same time, due to the nonlinearity of rocks, their elastic modulus and Poisson’s ratio are related to stress levels, but the in-situ stress value is unknown, so the iterative method is also required to solve the in-situ stress value to use the elastic modulus of rocks that is equivalent to the in-situ stress level. Hence, the double iteration method should be used to solve the rock elastic modulus value, Poisson’s ratio, correction coefficients, and in-situ stress value. This calculation method can ensure the accuracy of stress measurement results. The application of these technologies has significantly improved the accuracy of in-situ stress measurements, and the measurement accuracy in fractured and porous rock masses has increased by more than 20%.

### Measured stress data

To obtain accurate and reliable stress measurement data, the OC equipment, setup, and testing procedures adopted at the measuring points strictly follow the recommendations provided by the ISRM^31^. The accuracy of the OC stress measurements is not only influenced by the instrument itself and the measurement operations but also largely constrained by objective factors such as engineering geological environment and rock conditions. Therefore, the selection of in-situ stress measurement points should follow the following principles proposed: (1) the measurement points should avoid stress variation zones and be arranged in positions that are not affected by engineering excavation disturbances, namely, the original rock stress area, which requires that the depth of the borehole should reach at least 3–5 times the span of a tunnel; (2) the measuring points should be arranged in continuous and complete rock masses as far as possible, generally far away from faults, and away from rock fracture zones and fault development zones unless the measurements are intentionally carried out, to ensure the bonding quality between the hollow inclusion strain gauge and the borehole and obtain complete overcored rock cores, which is very necessary for the subsequent experiments and analysis of stress relief measurement results; (3) the measuring points shall be far away from or as far away as possible from larger excavation bodies, such as large goafs, large chambers, etc.; (4) to investigate the variation law of in-situ stress state with depth, the measurements should be carried out at least at three depth levels; (5) equipment and personnel needs have to be assessed; and (6) avoiding other potential interference sources that may affect stress measurements.

**Figure 3:**
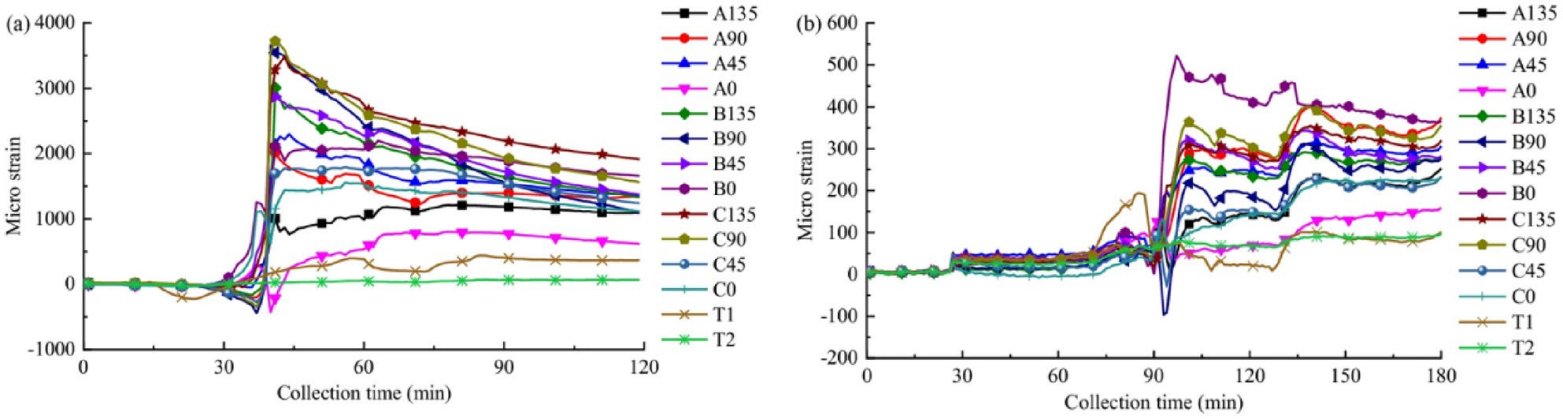
Typical stress relief curves^45^.

After the stress relief is completed, the strain data stored in the data collector are printed out by the computer, and the stress relief curve (i.e., the curve of the strain value of each strain gauge changing with the depth of borehole relief) is drawn accordingly. The stress relief curve is very helpful to check the working state of each strain gauge and determine whether the measured strain is affected by other factors besides stress relief, such as rock conditions. If each strain gauge is in normal working condition and the rock condition is good, the stress relief curve should show a regular and predictable shape (Fig. 3). In the rock core temperature calibration test, the additional strain value of each strain gauge caused by the temperature change of the measuring point in the stress relief process can be obtained by multiplying the temperature change value of the strain gauge during stress relief by the temperature strain rate. By removing this additional strain value from the final stable strain value measured during stress relief, the strain value really caused by stress relief can be derived.

Based on the stress relief strain values after temperature calibration and the results of confining pressure calibration tests, the *K* coefficients, rock elastic modulus, Poisson’s ratio, and in-situ stress values for each measurement point can be calculated using a self-developed double iteration calculation program, ensuring the correctness of the calculation process and results. In recent years, the improved OC technique has been employed by our team to conduct in-situ stress measurement campaigns in 14 mines (including eight metal mines and four coal mines) in China, and a total of 123 groups of OC stress data were obtained. Detailed information on the measured stress data is presented in Table 1. These stress data are distributed in eight provinces of China with different geological tectonic settings, and their measurement depth ranges from 56 to 1123 m, which has good representativeness. The absolute value of stress indicates that the present-day crustal stress state in these mining areas is compressed. The “direction” of stress is defined as 0° at due north, rotating clockwise, and 360° when turning to due north again. The positive or negative of the “dip” of stress is defined according to the horizontal plane, and it is positive when the horizontal plane angle is upward, and vice versa. Note that since the stress is a pair of collinear vectors, if a principal stress at a site lies in the NW–SE direction, its dip angle is positive from the fourth quadrant and negative from the second quadrant. To validate the quality of the obtained stress data and ensure the comparability of the data, the stress data are checked by using the globally accepted World Stress Map (WSM) quality ranking system^46^ regarding the OC method, and these data can be ranked as categories A, B, C, and D, revealing reliable and significant information on the in-situ stress tensor.

**Table 1:** Summary of the OC stress measurements in some mines in China.

Mine name	No	Depth (m)	Maximum principal stress (*σ*_1_)			Intermediate principal stress (*σ*_2_)			Minimum principal stress (*σ*_3_)			Quality
			Value (MPa)	Direction (°)	Dip (°)	Value (MPa)	Direction (°)	Dip (°)	Value (MPa)	Direction (°)	Dip (°)	
Sanshandao gold mine	1	75	6.01	288.50	− 6.30	3.81	198.00	− 4.90	2.56	250.40	82.00	A
	2	150	7.73	280.90	− 5.20	5.48	9.40	16.60	4.50	27.70	72.50	
	3	420	19.27	284.10	− 21.30	11.05	18.50	− 11.10	10.88	134.40	− 65.70	
	4	420	19.39	120.40	− 14.90	10.92	169.20	68.10	9.44	34.70	15.80	
	5	510	24.55	129.00	4.00	16.35	− 138.00	2.00	14.49	133.00	− 85.00	
	6	510	24.64	− 111.00	3.00	15.68	155.00	82.00	15.02	161.00	− 10.00	
	7	555	25.71	− 45.00	− 13.00	14.00	14.00	73.00	13.00	50.00	− 20.00	
	8	600	28.88	103.00	1.00	16.54	10.00	76.00	14.77	13.00	− 8.00	
	9	600	30.17	110.00	− 16.00	18.83	24.00	− 11.00	16.94	236.00	− 70.00	
	10	645	29.57	112.00	− 3.00	19.56	− 177.00	− 80.00	15.48	− 156.00	− 9.00	
	11	690	31.50	− 80.00	2.00	19.08	230.00	− 79.00	17.54	10.00	− 10.00	
	12	690	29.77	− 83.00	4.00	20.84	− 8.00	− 74.00	19.63	8.00	15.00	
	13	750	33.22	119.00	− 10.00	19.93	− 89.00	− 82.00	17.10	208.00	− 8.00	
	14	750	32.78	105.60	− 0.59	19.6	18.70	79.20	16.68	15.50	− 10.80	
	15	780	30.72	133.30	− 14.90	26.41	− 135.60	− 4.17	18.09	149.7	74.50	
	16	795	48.93	164.09	3.00	23.15	74.41	− 5.97	21.66	47.22	83.29	
	17	825	46.95	40.06	3.84	28.88	− 49.55	− 5.77	26.49	− 83.43	83.06	
	18	960	41.63	145.30	− 10.30	26.79	165.50	60.30	25.42	200.20	− 6.21	
Xincheng gold mine	1	205	11.45	307.10	− 17.60	5.69	286.30	71.30	4.03	35.10	6.20	A
	2	205	11.54	270.00	4.30	6.77	181.50	− 19.00	5.72	347.80	− 70.04	
	3	205	11.27	218.90	10.20	5.68	220.20	− 79.80	3.98	129.00	− 0.20	
	4	235	14.62	237.60	9.20	10.17	329.90	13.90	5.63	295.10	− 73.20	
	5	235	13.69	128.70	− 7.80	6.83	131.30	82.20	5.06	38.80	0.30	
	6	235	12.99	301.90	− 0.60	6.14	208.20	− 81.30	5.00	212.00	8.70	
	7	235	13.60	311.00	− 1.40	8.93	220.70	− 10.40	6.85	228.80	79.50	
	8	235	12.58	280.00	− 13.20	7.85	187.30	− 11.10	6.92	238.50	72.60	
	9	235	12.80	127.10	− 7.20	7.41	35.90	− 9.70	5.89	72.40	78.00	
	10	310	18.39	123.10	− 1.60	11.65	213.20	− 3.30	10.73	187.70	86.40	
	11	310	18.50	285.50	− 17.70	8.89	80.80	− 70.60	7.05	13.00	7.60	
	12	310	20.73	109.90	− 0.40	9.00	201.90	− 79.10	7.01	199.80	10.90	
	13	310	16.32	82.90	3.20	9.19	13.00	− 80.70	7.99	172.40	− 8.70	
	14	410	29.62	308.90	− 5.30	13.77	193.20	− 78.00	11.98	219.90	10.70	
	15	410	31.49	148.40	− 6.90	14.13	267.70	− 76.00	11.08	236.90	12.00	
	16	410	31.55	327.20	11.77	13.89	219.00	− 79.10	11.77	237.80	10.30	
	17	410	25.98	90.70	− 4.50	11.54	106.70	85.30	5.78	0.80	1.30	
	18	610	30.21	300.00	− 22.00	17.12	357.00	− 72.00	13.09	28.00	15.00	
	19	660	33.35	280.00	− 9.00	21.46	322.00	− 78.00	20.08	11.00	8.00	
	20	660	31.68	276.00	− 10.00	19.04	252.00	− 80.00	16.55	9.00	6.00	
Linglong gold mine	1	250	17.63	52.60	4.70	8.62	321.90	7.70	7.58	353.60	− 81.00	A
	2	250	14.06	287.7	− 14.40	7.63	19.40	− 6.60	6.63	133.50	− 74.10	
	3	290	15.58	141.40	− 3.00	8.28	24.50	− 83.30	6.84	51.80	5.90	
	4	290	17.51	294.80	− 0.10	9.37	26.30	− 84.30	7.26	24.80	5.70	
	5	290	17.68	280.30	− 13.50	9.25	322.80	72.00	6.61	193.20	11.70	
	6	290	20.45	343.50	− 6.40	8.36	75.30	− 15.10	7.75	51.20	73.50	
	7	290	19.74	91.30	− 2.10	10.09	171.90	77.10	8.58	1.80	12.70	
	8	370	23.43	138.20	− 9.30	12.69	12.70	− 74.20	10.13	50.30	12.60	
	9	370	21.32	103.60	− 12.00	10.68	237.40	− 72.90	8.20	103.60	− 12.00	
	10	410	25.77	255.70	2.60	10.73	155.40	75.60	10.18	166.40	− 14.10	
	11	410	25.55	218.00	2.10	11.51	118.80	77.10	8.64	128.50	− 12.70	
	12	570	32.53	92.20	− 3.80	15.54	199.00	− 77.00	13.21	181.40	12.40	
	13	920	53.13	134.70	− 5.30	27.72	81.40	81.20	25.51	44.10	− 7.00	
	14	920	55.88	128.10	− 3.80	30.12	229.20	− 71.20	28.41	216.00	10.70	
	15	920	50.17	273.30	− 15.80	27.72	314.10	70.20	24.89	187.00	13.10	
	16	970	60.26	335.00	11.00	34.52	34.00	72.20	27.93	246.00	− 13.10	
	17	970	57.92	136.10	− 0.50	30.24	227.10	− 70.00	26.96	226.00	15.00	
	18	970	57.22	295.20	10.40	28.90	205.10	3.50	28.52	36.30	80.00	
Shuichang iron mine	1	81	4.07	272.20	− 7.30	2.38	3.90	− 13.30	2.16	154.20	− 74.80	D
	2	91.5	4.26	90.60	− 0.80	2.86	180.60	− 2.90	2.68	344.90	− 87.00	
	3	56	3.68	98.90	− 7.20	2.33	189.70	− 6.20	2.03	319.80	80.50	
Pingdingshan No. 1 mine	1	440	19.03	111.20	12.20	12.66	49.40	− 65.30	11.40	196.40	− 21.10	C
	2	490	18.64	180.80	1.50	15.12	88.30	60.00	14.39	91.70	− 29.90	
	3	555	28.13	149.70	− 5.90	19.06	− 12.70	− 83.80	15.26	239.90	− 1.90	
	4	556	19.74	98.10	53.30	16.75	39.70	− 21.30	14.83	141.80	− 28.30	
	5	633	22.06	120.70	− 1.10	17.63	31.40	36.40	14.99	209.20	53.60	
	6	652	22.39	170.20	− 14.30	17.65	79.30	− 3.70	14.20	155.20	75.20	
	7	692	30.83	109.60	9.70	14.68	35.10	− 57.30	12.85	193.70	− 30.80	
Pingdingshan No. 8 mine	1	417	13.74	282.10	− 16.80	11.61	191.60	− 1.90	7.91	95.40	− 73.10	C
	2	417	14.62	107.70	− 20.30	11.35	197.80	− 0.30	7.23	288.60	− 69.70	
	3	523	16.70	332.70	− 18.30	12.25	238.40	− 12.70	9.09	115.50	− 67.50	
	4	524	16.72	332.40	− 18.50	12.25	238.20	− 12.40	9.07	116.20	− 67.50	
	5	562	18.29	288.60	− 1.90	10.82	198.20	− 11.40	8.38	208.20	78.50	
Pingdingshan No. 10 mine	1	514	31.43	53.20	6.10	17.48	131.10	− 72.60	15.44	146.20	16.10	B
	2	514	29.30	229.10	− 6.80	18.34	137.40	− 16.90	17.10	160.20	72.20	
	3	785	34.32	202.40	− 16.90	22.19	219.00	71.40	18.29	293.40	− 4.80	
	4	793	36.19	60.30	15.00	25.07	49.30	− 73.60	19.07	330.10	3.40	
	5	869	44.40	56.00	− 13.60	25.48	333.30	− 11.50	17.18	21.50	70.40	
	6	869	44.28	61.50	− 5.60	26.09	330.30	− 8.80	18.45	6.10	79.40	
	7	914	40.20	43.10	− 7.80	28.27	132.20	2.30	14.24	27.50	81.40	
	8	914	43.36	229.2	9.30	23.12	133.00	− 4.50	16.42	42.00	79.10	
	9	1061	43.06	228.10	13.30	26.10	60.50	76.20	22.36	137.50	− 2.50	
	10	1061	44.06	60.40	− 1.80	28.38	155.30	71.60	24.20	149.10	17.50	
	11	1123	65.46	60.10	− 1.00	38.06	209.40	− 75.80	31.26	149.20	15.30	
Meishan iron mine	1	210	9.46	228.10	2.00	4.36	318.50	11.70	3.04	308.60	− 78.10	B
	2	210	11.79	140.20	− 5.70	5.86	48.90	− 13.10	5.03	73.20	75.70	
	3	218	11.47	359.50	− 4.50	5.61	270.30	9.90	4.80	65.20	79.10	
	4	342	20.19	107.30	− 3.10	9.79	114.70	86.90	7.48	17.30	0.40	
	5	350	20.32	195.90	12.60	11.79	287.30	6.20	9.57	43.00	76.00	
	6	350	18.37	335.10	− 4.50	9.57	307.20	85.20	6.92	64.90	2.40	
	7	360	16.16	345.50	− 0.30	10.28	211.30	− 89.50	7.59	255.50	0.30	
	8	418	21.50	313.70	− 3.30	12.32	193.30	− 83.50	11.56	224.10	5.60	
Datong coal mine area	1	352	12.95	330.70	0.50	7.29	56.70	− 83.00	7.14	60.70	7.30	D
	2	360	12.36	324.90	3.20	6.91	164.80	86.60	6.37	55.00	1.10	
	3	364	12.05	325.00	− 14.50	7.50	310.00	75.00	5.83	54.00	3.70	
	4	370	13.11	331.70	− 1.80	11.04	241.40	− 7.50	8.74	255.00	82.30	
Lilou iron mine	1	300	10.99	60.34	3.94	4.44	18.81	84.74	4.18	330.10	3.47	C
	2	350	21.21	194.11	− 5.03	6.69	305.70	− 76.53	3.84	283.00	12.46	
	3	350	15.42	167.76	2.98	6.89	76.92	15.61	6.09	88.28	− 74.09	
	4	350	17.31	38.03	− 0.26	8.25	307.46	− 65.43	7.09	308.16	24.57	
	5	400	14.65	8.31	7.77	12.07	246.38	75.53	7.83	280.00	− 12.13	
	6	400	24.16	179.16	2.10	12.08	257.14	− 80.01	9.07	269.53	9.76	
Ekou iron mine	1	310	23.10	359.90	− 2.00	7.64	89.90	1.10	9.41	331.40	87.70	D
	2	310	23.11	170.00	− 0.50	8.25	258.10	74.50	10.72	260.20	− 15.50	
	3	310	22.96	183.20	0.80	8.85	93.40	− 15.60	10.97	90.50	74.30	
	4	310	19.34	153.10	− 0.30	8.57	64.60	78.00	8.99	63.00	− 12.00	
Jinchuan No.2 mine	1	580	31.18	33.80	6.30	13.74	280.90	74.10	10.88	305.40	− 14.50	B
	2	580	24.88	1.90	15.50	13.59	271.30	2.10	12.96	353.60	− 74.40	
	3	580	28.08	35.20	5.00	14.28	88.70	− 82.70	11.59	305.80	− 6.70	
	4	580	28.44	36.60	2.20	13.34	299.40	72.90	9.44	307.20	− 16.90	
	5	730	36.95	176.70	− 8.80	17.55	2.60	− 81.10	13.09	86.80	0.90	
	6	730	37.86	18.20	1.40	16.79	130.60	86.20	12.22	108.20	− 3.50	
	7	730	34.68	348.00	− 5.10	17.34	238.60	− 74.90	13.48	259.20	14.20	
	8	730	31.64	13.20	3.80	18.68	79.90	− 80.50	11.59	283.80	− 8.70	
	9	790	40.55	160.60	− 1.90	20.55	0.30	− 84.30	16.75	70.60	0.70	
	10	790	37.26	226.00	14.60	18.19	204.20	− 74.50	17.66	314.60	− 5.60	
Beiminghe iron mine	1	891	21.99	92.30	− 0.60	10.55	174.10	85.50	9.14	2.30	4.50	C
	2	941	23.12	332.80	− 2.00	15.64	242.80	− 1.20	12.45	121.10	− 87.60	
	3	933	24.72	295.90	− 5.90	15.07	205.50	− 4.00	13.23	261.80	82.90	
	4	984	25.60	282.50	− 1.10	14.48	189.10	72.70	12.32	192.80	17.20	
	5	956	31.60	117.90	− 10.40	20.64	209.00	− 6.20	17.92	329.20	− 77.90	
Gongchangling iron mine	1	355	9.30	69.20	− 3.30	8.50	47.70	86.50	6.50	− 20.90	− 1.30	D
	2	405	12.60	− 109.30	− 10.50	11.20	− 85.30	82.60	4.60	− 23.20	− 7.40	
	3	405	13.40	− 111.30	− 12.60	12.90	− 96.90	77.00	5.20	− 20.60	− 3.10	
	4	494	12.30	80.30	3.80	10.10	9.30	− 78.40	7.00	169.60	− 10.90	

**Figure 4:**
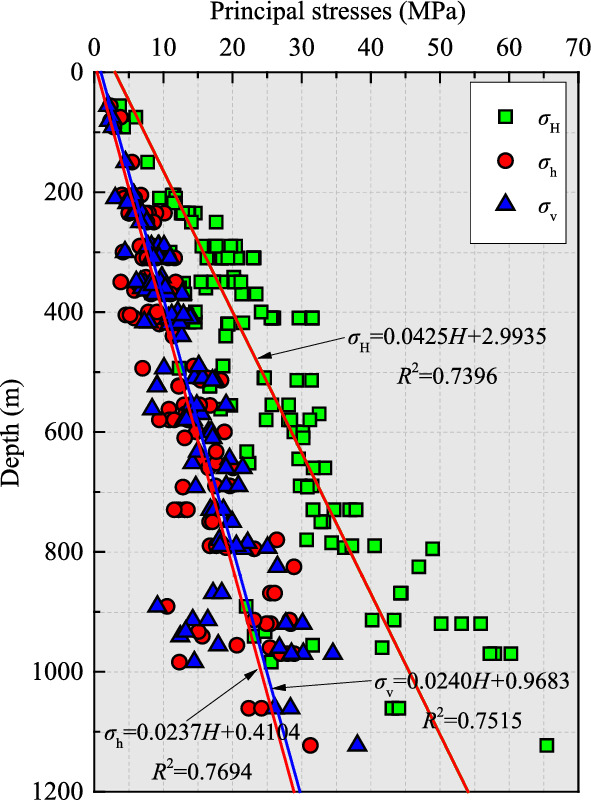
Constrained principal stress profile with depth.

As shown in Table 1, although these stress data are distributed in different regions with distinct tectonic settings, the measurement results indicate that the maximum principal stress at each measuring point is close to the horizontal direction (called *σ*_H_). For the remaining two principal stresses, i.e., intermediate and minimum principal stresses, one is nearly horizontal (called *σ*_h_) and the other remains basically vertical (called *σ*_v_). This reflects that horizontal tectonic stress plays a dominant role in the stress field of those mining areas, which matches more or less with the first-order pattern of the crustal stress in the lithosphere^47^. Hence, the derived OC stress data agree with the hypothesis that horizontal tectonic stress normally regulates the modern crustal stress field of the upper crust. Moreover, the vertical principal stress magnitude is basically equal to or slightly less than the weight of overlying strata, and the magnitude of the maximum horizontal principal stress is around twice that of the vertical principal stress on average. In addition, the three principal stress magnitudes versus depth are plotted in Fig. 4, showing the correlation between the stress values and depth obtained through linear fitting analysis. The results indicate that the three principal stresses exhibit small dispersion and increase approximately linearly with depth, which is completely in accord with the current universal cognition of in-situ stress. On the other hand, the dominant direction of the maximum principal stress that we identified in each mine is in good agreement with the directions of various stress indicators previously derived from different measurement methods (such as OC, hydraulic fracturing, borehole slotter, and geological indicators) in and around the mining area in the recently updated WSM^48^ and the tectonic stress map of North China^49^. The above results suggest that the improved OC technique has excellent testing performance and reliable measurement results. The stress data has provided the necessary basis for the scientific design and construction of these mines, especially for the selection of appropriate roadway and stope location and direction, the optimal shape and size of underground openings, effective and safe excavation sequence, and reliable support and reinforcement of mining structures.

## In-situ stress prediction approach

### Grey model

**Figure 5:**
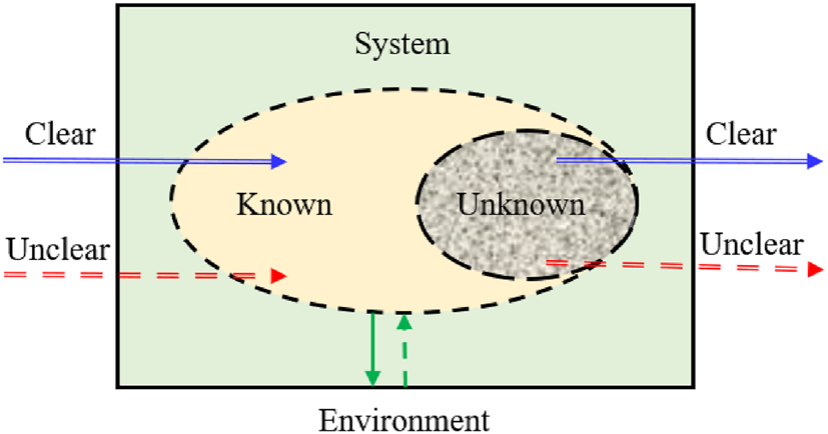
Concept diagram of the grey system.

The grey system theory was first proposed by Deng^50^ in the late 1970s, which mainly aimed at uncertainty problems with little experience and data. The information in the grey system includes both known and unknown, and there are uncertain relationships between various factors within the system (Fig. 5). Grey model (GM) is a prediction approach to establish a mathematical model and make a forecast through a small amount of incomplete information. In recent years, the grey prediction theory has been widely used in many fields. It can effectively deal with systems with significant uncertainties and limited data samples and exhibits noticeable advantages over traditional prediction approaches, so it is increasingly being applied. The essence of the grey theory is to weaken the randomness of the original random sequence by the method of generating information so that the original data sequence can be transformed into a new sequence that is easy to model. The model of the original grey sequence can be obtained by performing the corresponding inverse generation on the model built according to the new sequence.

Preprocessing the original data is required when establishing a grey model. In this study, the accumulating generation operator (AGO) is used for preprocessing, which sequentially accumulates the data in the original sequence to yield a new sequence. Its mathematical form is denoted as

7 $$X^{(1)} = AGO \bullet X^{(0)}$$

in which,

8 $$X^{(1)} = (x^{(1)} (1),x^{(1)} (2), \cdots ,x^{(1)} (n))$$

9 $$x^{(1)} (k) = \sum\limits_{m = 1}^{k} {x^{(0)} (m)} { (}k = 1,2, \cdots ,n)$$

By constructing data matrixs and vectors, the model parameters can be solved using the least square method. Assuming $x_{1}^{(0)} = (x_{1}^{(0)} (1),x_{1}^{(0)} (2), \cdots ,x_{1}^{(0)} (n))$ is a system characteristic data sequence, and

10 $$\left\{ \begin{gathered} x_{2}^{(0)} = (x_{2}^{(0)} (1),x_{2}^{(0)} (2), \cdots ,x_{2}^{(0)} (n)) \hfill \\ x_{3}^{(0)} = (x_{3}^{(0)} (1),x_{3}^{(0)} (2), \cdots ,x_{3}^{(0)} (n)) \hfill \\ \cdots \hfill \\ x_{N}^{(0)} = (x_{N}^{(0)} (1),x_{N}^{(0)} (2), \cdots ,x_{N}^{(0)} (n)) \hfill \\ \end{gathered} \right.$$

is the correlation factor sequence, and $x_{i}^{(1)}$ ($i = 1,2, \cdots ,N$) is the 1-AGO sequence of $x_{i}^{(0)}$, thus

11 $$x_{1}^{(1)} (k) = a + b_{2} x_{2}^{(1)} (k) + b_{3} x_{3}^{(1)} (k) + \cdots + b_{N} x_{N}^{(1)} (k)$$

is called the GM(0, N) model.

The GM(0, N) model does not contain derivatives, so it is a static model. It looks like a multivariate linear regression model, but it is essentially different from the general multivariate linear regression model. The general modeling of multiple linear regression is based on the original data sequence, while the modeling basis of GM(0, N) is the 1-AGO sequence of the original data.

Assuming that $x_{i}^{(0)}$ and $x_{i}^{(1)}$ meet the above definitions, let the parameter columns be

12 $$u = [a,b_{2} ,b_{3} , \cdots ,b_{N} ]^{T}$$

13 $$B = \left[ {\begin{array}{*{20}c} {x_{2}^{(1)} (2)} & {x_{3}^{(1)} (2)} & \cdots & {x_{N}^{(1)} (2)} \\ {x_{2}^{(1)} (3)} & {x_{3}^{(1)} (3)} & \cdots & {x_{N}^{(1)} (3)} \\ \vdots & \vdots & \ddots & \vdots \\ {x_{2}^{(1)} (n)} & {x_{3}^{(1)} (n)} & \cdots & {x_{N}^{(1)} (n)} \\ \end{array} } \right]$$

14 $$Y = \left[ {\begin{array}{*{20}c} {x_{1}^{(1)} (2)} \\ {x_{1}^{(1)} (3)} \\ {} \\ {x_{1}^{(1)} (n)} \\ \end{array} } \right]$$

The residual vector is

15 $$E = Y - Bu$$

When $E^{T} E \to \min$, the model can fit the AGO sequence well. Define the objective function as

16 $$J = E^{T} E = (Y - Bu)^{T} (Y - Bu)$$

Expanding Eq. (16) yields:

17 $$J = Y^{T} Y - 2(Bu)^{T} Y + u^{T} B^{T} Bu$$

The minimum value of the objective function is at the point where the derivative of the parameter column *u* is zero, so there is:

18 $$\nabla J = 2B^{T} Bu - 2B^{T} Y = 0$$

According to the least square technique, it can be estimated that:

19 $$u = (B^{T} B)^{ - 1} B^{T} Y$$

Hence, the GM(0, N) model of the AGO sequence is obtained. By performing a reverse generation on this model, the model of the original sequence can be derived. If the regularity of the sequence $x^{(1)}$ is not strong, a 2-AGO sequence $x^{(2)}$ can be generated until a $\xi$-AGO sequence $x^{(\xi )}$. In this case, the established model can be inversely generated $\xi$ times to determine the original sequence model.

Based on the measured in-situ stress data of each measuring point, the *σ*_H_, *σ*_h_, and *σ*_v_ sequences and the depth *z* sequences of each measuring point are respectively combined as known grey sequences to establish the GM(0, 1) model:

20 $$\sigma_{i} = a + bz$$

where *σ*_*i*_ represents *σ*_H_, *σ*_h_, or *σ*_v_; and *a* is the undetermined constant of the model.

The accuracy of the model is a true reflection of the correctness and practicability of the model prediction. The relative error size test method is employed in this study to check whether the model is reasonable. This method is an intuitive and point-by-point comparison arithmetic test method, which compares the predicted data with the actual data to observe whether the relative error meets the practical requirements.

Assuming the prediction result of the GM(0, 1) model is $\hat{x}_{1}^{(0)} = (\hat{x}_{1}^{(0)} (1),\hat{x}_{1}^{(0)} (2), \cdots ,\hat{x}_{1}^{(0)} (n))$, the residual can be calculated to obtain:

21 $$E = (e(1),e(2), \cdots ,e(n)) = x_{1}^{(0)} - \hat{x}_{1}^{(0)}$$

where $$e(k) = x_{1}^{(0)} (k) - \hat{x}_{1}^{(0)} (k) \, (k = 1,2, \cdots ,n)$$.

The relative error is:

22 $$rel(k) = \frac{e(k)}{{x_{1}^{(0)} (k)}} \times 100\% ,k = 1,2, \cdots ,n$$

The average relative error is:

23 $$rel = \frac{1}{n}\sum\limits_{k = 1}^{n} {\left| {rel(k)} \right|}$$

The accuracy of the GM(0, 1) model is given by

24 $$p^{o} = (1 - rel) \times 100\%$$

Successful modeling generally requires $p^{o} > 80\%$, preferably $p^{o} > 90\%$.

**Figure 6:**
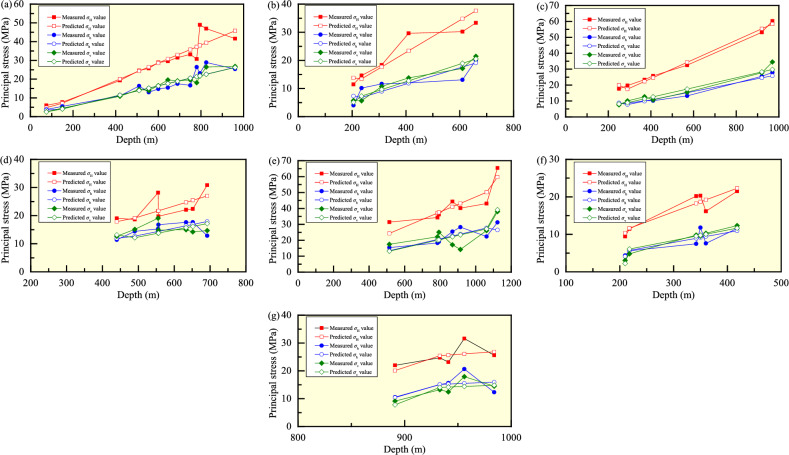
Comparison between the predicted values using the GM model and measured values of the three principal stresses in the Sanshandao gold mine (**a**), Xincheng gold mine (**b**), Linglong gold mine (**c**), Pingdingshan No. 1 mine (**d**), Pingdingshan No. 10 mine (**e**), Meishan iron mine (**f**), and Beiminghe iron mine (**g**).

Based on the measured in-situ stress data (Table 1), seven mines, namely Sanshandao gold mine, Xincheng gold mine, Linglong gold mine, Pingdingshan No. 1 mine, Pingdingshan No. 10 mine, Meishan iron mine, and Beiminghe iron mine, have a large amount of stress data, which are selected as training samples. Note that for the stress data at the same depth in a mine, one set of data is chosen as the representative data. Grey prediction model GM(0, 1) is used to predict the magnitudes of *σ*_H_, *σ*_h_, and *σ*_v_ at each measuring point in these seven mines, and the results are listed in Table 2 and the intuitive comparison between the predicted values and measured values is plotted in Fig. 6. In the seven mines, the mean relative errors of the predicted *σ*_H_ values are 9.4446%, 13.6101%, 6.6579%, 11.3630%, 10.6638%, 8.4339%, and 8.9955%, respectively; the mean relative errors of the predicted *σ*_h_ values are 13.5686%, 29.7263%, 6.9395%, 12.4404%, 14.2287%, 13.1935%, and 11.7173%, respectively; and the mean relative errors of the predicted *σ*_v_ values are 6.1479%, 12.1361%, 10.7058%, 13.3870%, 21.9825%, 10.8826%, and 11.3876%, respectively. Obviously, the mean relative errors of the prediction results of GM(0, 1) for the three principal stresses all reach 6–30%, and the accuracy of the model fails to meet the requirements, which means that the reliability of the prediction results is not high. Thus, the BPNN with strong prediction and fitting ability is considered to achieve higher prediction accuracy.

**Table 2:** Predicted stress values and mean relative errors using GM(0, 1) in the seven mines.

Mine name	Depth (m)	*σ*_H_				*σ*_h_				*σ*_v_			
		MV (MPa)	PV (MPa)	MRE (%)	RMSE (MPa)	MV (MPa)	PV (MPa)	MRE (%)	RMSE (MPa)	MV (MPa)	PV (MPa)	MRE (%)	RMSE (MPa)
Sanshandao gold mine	75	6.01	4.78	9.4446	4.3963	3.81	3.46	13.5686	2.8367	2.56	2.51	6.1479	1.5941
	150	7.73	7.16			5.48	4.11			4.50	4.10		
	420	19.27	20.04			11.05	11.50			10.88	11.49		
	510	24.55	24.33			16.35	13.97			14.49	13.95		
	555	25.71	26.48			13.00	15.20			14.00	15.18		
	600	28.88	28.63			14.77	16.43			16.54	16.41		
	645	29.57	30.77			15.48	17.67			19.56	17.64		
	690	31.50	32.92			17.54	18.90			19.08	18.87		
	750	33.22	35.78			16.68	20.54			19.60	20.51		
	780	30.72	37.21			26.41	21.36			18.09	21.33		
	795	48.93	37.93			23.15	21.77			21.66	21.74		
	825	46.95	39.36			28.88	22.60			26.49	22.56		
	960	41.63	45.80			25.42	26.29			26.79	26.25		
Xincheng gold mine	205	11.45	13.79	13.6101	3.7827	4.03	7.32	29.7263	2.9218	5.69	5.11	12.1361	1.2683
	235	14.62	13.41			10.17	6.80			5.63	7.30		
	310	18.39	17.69			11.65	8.97			10.73	9.63		
	410	29.62	23.40			11.98	11.87			13.77	12.73		
	610	30.21	34.81			13.09	17.66			17.12	18.94		
	660	33.35	37.66			20.08	19.10			21.46	20.50		
Linglong gold mine	250	17.63	19.92	6.6579	1.8793	8.62	8.83	6.9395	1.2307	7.58	8.11	10.7058	2.1782
	290	19.74	17.45			8.58	7.73			10.09	8.92		
	370	23.43	22.27			10.13	9.87			12.69	11.37		
	410	25.77	24.67			10.18	10.93			10.73	12.60		
	570	32.53	34.30			13.21	15.20			15.54	17.52		
	920	53.13	55.36			25.51	24.53			27.72	28.28		
	970	60.26	58.37			27.93	25.86			34.52	29.82		
Pingdingshan No. 1 mine	440	19.03	17.87	11.3630	3.3447	11.40	11.89	12.4404	2.3073	12.66	13.14	13.3870	2.6388
	490	18.64	19.12			14.39	12.68			15.12	12.15		
	652	22.39	25.44			17.65	16.87			14.20	16.17		
	692	30.83	27.00			12.85	17.90			14.68	17.16		
	633	22.06	24.70			17.63	16.38			14.99	15.69		
	556	19.74	21.69			16.75	14.38			14.83	13.79		
	555	28.13	21.65			15.26	14.36			19.06	13.76		
Pingdingshan No. 10 mine	1123	65.46	59.68	10.6638	4.8401	31.26	26.47	14.2287	3.5960	38.06	39.19	21.9825	4.7441
	1061	43.06	50.15			22.36	27.75			26.10	27.14		
	785	34.32	37.11			18.29	20.53			22.19	20.08		
	793	36.19	37.48			19.07	20.74			25.07	20.28		
	869	44.40	41.08			25.48	22.72			17.18	22.23		
	514	31.43	24.30			15.44	13.44			17.48	13.15		
	914	40.20	43.20			28.27	23.90			14.24	23.38		
Meishan iron mine	210	9.46	10.30	8.4339	1.6901	4.36	4.10	13.1935	1.4656	3.04	2.24	10.8826	0.6916
	218	11.47	11.63			5.61	5.71			4.80	6.08		
	342	20.19	18.24			7.48	8.95			9.79	9.54		
	350	20.32	18.67			11.79	9.16			9.57	9.76		
	360	16.16	19.20			7.59	9.42			10.28	10.04		
	418	21.50	22.30			11.56	10.94			12.32	11.66		
Beiminghe iron mine	891	21.99	20.10	8.9955	2.9188	10.55	10.41	11.7173	2.8203	9.14	7.80	11.3876	1.8962
	941	23.12	25.68			15.64	15.25			12.45	14.19		
	933	24.72	25.46			15.07	15.12			13.23	14.07		
	984	25.60	26.85			12.32	15.95			14.48	14.84		
	956	31.60	26.09			20.64	15.50			17.92	14.41		

*MV* Measured value, *PV* Predicted value, *MRE* Mean relative error, and *RMSE* Root mean square error.

### Back propagation neural network

Back propagation neural network (BPNN), designed by Rumelhart et al.^51^ in 1986, is a multi-layer feedforward neural network based on error back propagation. It is the most popular type of neural network model and can achieve arbitrary nonlinear mapping between the input and output, which makes it widely used in fields such as function recognition and pattern recognition. The BPNN is a typical multi-layer network, which is divided into an input layer, hidden layer, and output layer. The connection mode between layers is that the input of the layer *i* is only connected with the output of layer *i*-1, and each neuron accepts the input of the previous layer and outputs it to the next layer without feedback. The input layer of the neural network takes values corresponding to the attributes of each training sample and assigns them to the input layer units. The outputs of these units are combined with corresponding weights and transmitted to the hidden layer units simultaneously. The weighted output of the hidden layer is then passed as input to the next hidden layer, and the weighted output of the last hidden layer node is passed to the output layer unit, which ultimately gives the predicted output of the corresponding sample. As long as there are enough hidden layers in the middle, the nonlinear threshold function in the multilayer forward network can fully approximate any function.

**Figure 7:**
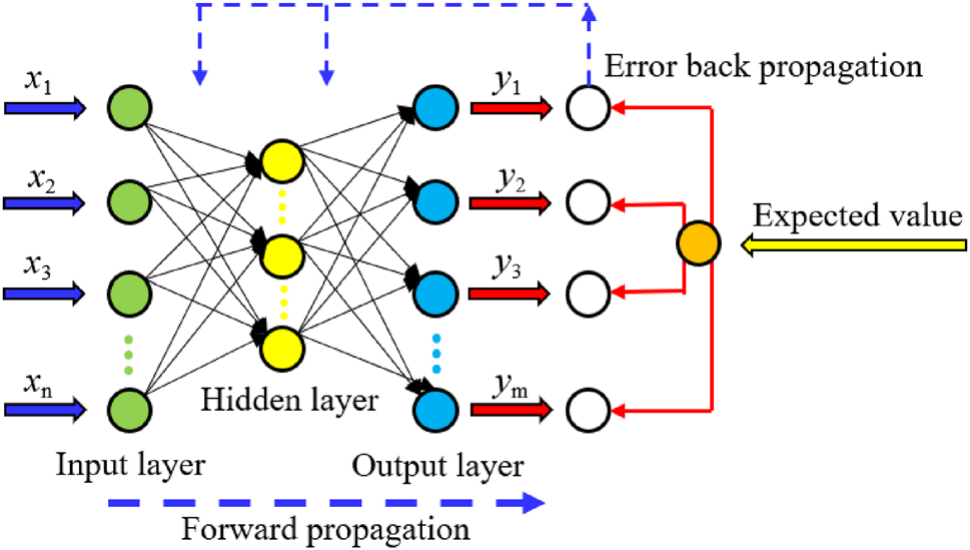
BPNN infrastructure.

The BPNN algorithm is the training algorithm for acyclic multilevel networks. Its basic idea is a gradient descent method, which uses the gradient search technique to minimize the mean square error between the actual output value and the expected output value of the network. The network learning process actually includes two stages: forward propagation and backward propagation, as illustrated in Fig. 7. In the forward propagation process, the input information is processed layer by layer from the input layer through the hidden layer and transmitted to the output layer, and the state of each layer of neurons only affects the state of the next layer of neurons. If the expected output cannot be obtained at the output layer, the error signal will be returned according to the original connection path, and the goal of minimizing the error signal will be achieved by modifying the weights of neurons at each layer.

Here, the implementation steps of a three-layer BPNN learning algorithm are provided, and so on for multi-layer situations.

1. Initialization. Assign random numbers to all neurons in the network in the interval of (− 1, 1).
2. Given training data samples: input vector $X = (x_{1} ,x_{2} , \cdots ,x_{n} )$ and expected output vector $Y = (y_{1} ,y_{2} , \cdots ,y_{m} )$.
3. Calculate the output layer by layer starting from the input layer.

All neurons in the input layer: the input is $x_{i}$, the output is $o_{i} = x_{i} \, (i = 1,2, \cdots ,n)$, where *n* is the number of neurons in the input layer.

All neurons in the hidden layer: the input is $x_{k} = \sum\nolimits_{i = 1}^{l} {w_{ik} o_{i} + } b_{k}$, the output is $o_{k} = f(x_{k} ) \, (k = 1,2, \cdots ,l)$, where *l* is the number of neurons in the hidden layer, $w_{ik}$ represents the weight of the *i*th neuron in the input layer to the *k*th neuron in the hidden layer, and $b_{k}$ denotes the offset of the *k*th neuron in the hidden layer.

All neurons in the output layer: the input is $x_{j} = \sum\nolimits_{k = 1}^{l} {w_{kj} o_{k} + } b_{j}$, the output is $o_{j} = g(x_{j} ) \, (j = 1,2, \cdots ,m)$, where *m* is the number of neurons in the output layer, $w_{kj}$ represents the weight from the *k*th neuron in the hidden layer to the *j*th neuron in the output layer, and $b_{j}$ represents the offset of the *j*th neuron in the output layer.

The functions $f( \cdot )$ and $g( \cdot )$ are usually called the excitation function and are typically the following Sigmoid function:

25 $$f(x) = \frac{1}{{1 + e^{ - x} }}$$

(4) Error back propagation, adjusting weights layer by layer from the output node.

Define the network error function:

26 $$E = \frac{1}{2}\sum\limits_{j = 1}^{m} {(o_{j} - y_{j} )^{2} }$$

Weight adjustment using gradient algorithm:

27 $$w(t + 1) = w(t) - \eta \nabla E(t)$$

where $- \eta \nabla E(t)$ is the opposite direction of the gradient change of the error function during *t* training.

The adjustment amount of the weight $w_{kj}$ from the output layer to the hidden layer is:

28 $$w_{kj} (t + 1) = w_{kj} (t) + \eta (y_{j} - o_{j} )o_{j} (1 - o_{j} )o_{k}$$

where $\eta$ is the learning rate, which is a positive constant, and $y_{j} { (}j = 1,2, \cdots ,m{)}$ is the expected output.

The adjustment amount of the weight $w_{ik}$ from the hidden layer to the input layer is:

29 $$w_{ik} (t + 1) = w_{ik} (t) + \eta o_{k} (1 - o_{k} )o_{i} \sum\limits_{j = 1}^{m} {\delta_{j} w_{kj} }$$

where $\delta_{j} = (y_{j} - o_{j} )o_{j} (1 - o_{j} )$, which represents the error of the *j*th neuron in the output layer.

(5) After the weight adjustment is completed, return to step (3) and recalculate until the error meets the requirements or the termination condition is reached.
  The BPNN can realize highly nonlinear mapping from N-dimensional space to M-dimensional space, which can better fit data sequences, deal with complex nonlinear problems, and have certain generalization abilities. However, the BPNN has many problems to be solved urgently, such as excessive iterations, slow convergence speed, and easy falling into local minimum. Given these problems, combined with the improved methods already proposed, the following new improvements have been made in this study:
(6) Adjust the activation function. The advantage of choosing the ReLU (Eq. (30)) function as the activation function is that neurons only need to judge whether the input is greater than 0, which is more efficient and faster in calculation. Compared to the saturation at both ends of the Sigmoid function, the ReLU function is a left saturation function, and its derivative is 1 when $x > 0$, which alleviates the problem of gradient elimination of neural networks to some extent and accelerates the convergence speed of gradient descent.
  30 $${\text{ReLU(}}x) = \left\{ {\begin{array}{*{20}c} {x{\text{ if }}x > 0} \\ {0{\text{ if }}x \le 0} \\ \end{array} } \right. = \max (0,x)$$
(7) Adaptive moment estimation (Adam) algorithm is selected to optimize and adjust the weights between networks. The gradient descent algorithm always maintains a single learning rate to update all weights, and the learning rate will not change with the progress of training. The Adam algorithm designs independent adaptive learning rates for different parameters by calculating the first-order moment estimation and the second-order moment estimation of gradients, thereby better balancing the convergence speed and stability. Meanwhile, the adaptive learning rate mechanism and second-order moment estimation of the Adam algorithm are helpful in preventing gradient explosion and vanishing problems. The main calculation process of this algorithm is as follows:
(8) Initialization: Given the learning rate $\eta$, smoothing constants $\beta_{1}$ and $\beta_{2}$, and initial network weights, i.e., $m_{0} = 0$ and $v_{0} = 0$.
(9) Calculating gradient $g_{t}$: the first-order moment estimation of the gradient $m_{t} = \beta_{1} m_{t - 1} + (1 - \beta_{1} )g_{t}$ and the second-order moment estimation of the gradient $v_{t} = \beta_{2} v_{t - 1} + (1 - \beta_{2} )g_{t}$, and correct deviations $\hat{m}_{t} = \frac{{m_{t} }}{{1 - \beta_{1}^{t} }}$ and $\hat{v}_{t} = \frac{{v_{t} }}{{1 - \beta_{2}^{t} }}$.
(10) Updating parameters: $$\theta_{t} = \theta_{t - 1} - \eta \frac{{\hat{m}_{t} }}{{\sqrt {\hat{v}_{t} } + \varepsilon }}$$.

**Figure 8:**
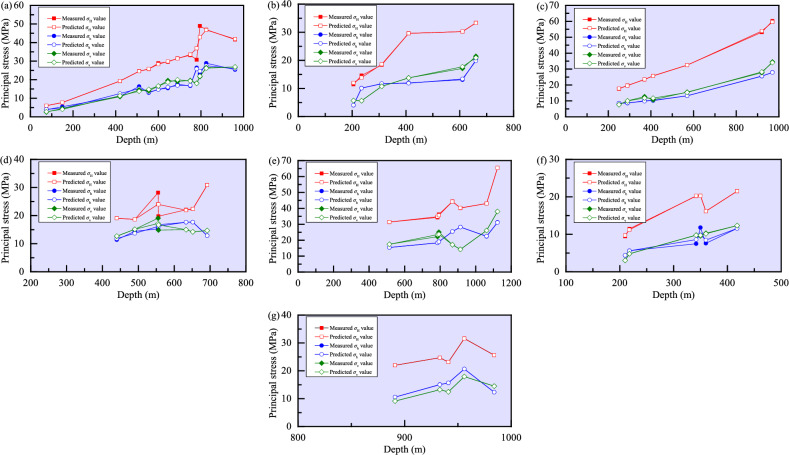
Comparison between the predicted values using the BPNN model and measured values of the three principal stresses in the Sanshandao gold mine (**a**), Xincheng gold mine (**b**), Linglong gold mine (**c**), Pingdingshan No. 1 mine (**d**), Pingdingshan No. 10 mine (**e**), Meishan iron mine (**f**), and Beiminghe iron mine (**g**).

According to the measured in-situ stress data in the seven mines, a single-input single-output BPNN model is adopted. That is, the number of neurons in the input layer is 1, the number of neurons in both hidden layers is 10, and the number of neurons in the output layer is 1, and the learning rate is 0.02. After the training, the predicted values and average relative errors of the three principle tresses using the model are presented in Table 3 and their comparison is shown in Fig. 8. In the seven mines, the mean relative errors of the predicted *σ*_H_ values are 2.9863%, 1.8531%, 0.6831%, 5.2761%, 0.4801%, 0.9130%, and 0.0169%, respectively; the mean relative errors of the predicted *σ*_h_ values are 5.5816%, 1.1250%, 1.1399%, 2.5183%, 0.3028%, 7.2294%, and 0.1274%, respectively; and the mean relative errors of the predicted *σ*_v_ values are 3.6198%, 1.0315%, 3.2713%, 3.2308%, 1.6501%, 0.5832%, and 0.0125%, respectively. It can be observed that the average relative errors of the three principal stresses are all less than 10% (0.0125–7.2294%), and the model accuracy meets the requirements and has sufficient credibility.

**Table 3:** Predicted stress values and mean relative errors based on the BPNN in the seven mines.

Mine name	Depth (m)	*σ*_H_				*σ*_h_				*σ*_v_			
		MV (MPa)	PV (MPa)	MRE (%)	RMSE (MPa)	MV (MPa)	PV (MPa)	MRE (%)	RMSE (MPa)	MV (MPa)	PV (MPa)	MRE (%)	RMSE (MPa)
Sanshandao gold mine	75	6.01	6.02	2.9863	2.4142	3.81	4.07	5.5816	1.3355	2.56	2.77	3.6198	0.5759
	150	7.73	7.71			5.48	4.49			4.50	4.05		
	420	19.27	19.25			11.05	12.56			10.88	11.35		
	510	24.55	24.50			16.35	15.25			14.49	13.78		
	555	25.71	25.84			13.00	13.30			14.00	14.75		
	600	28.88	28.31			14.77	14.67			16.54	16.58		
	645	29.57	29.82			15.48	15.99			19.56	18.66		
	690	31.50	31.40			17.54	16.86			19.08	19.96		
	750	33.22	33.51			16.68	17.02			19.60	19.30		
	780	30.72	36.91			26.41	25.83			18.09	18.01		
	795	48.93	42.86			23.15	24.59			21.66	21.94		
	825	46.95	46.76			28.88	27.52			26.49	26.18		
	960	41.63	41.89			25.42	25.88			26.79	26.90		
Xincheng gold mine	205	11.45	11.98	1.8531	0.3859	4.03	4.07	1.1250	0.1696	5.69	5.69	1.0315	0.3305
	235	14.62	13.87			10.17	10.08			5.63	5.63		
	310	18.39	18.61			11.65	11.72			10.73	10.73		
	410	29.62	29.61			11.98	11.87			13.77	13.75		
	610	30.21	30.24			13.09	13.36			17.12	17.74		
	660	33.35	33.32			20.08	19.81			21.46	20.94		
Linglong gold mine	250	17.63	17.75	0.6831	0.4049	8.62	8.57	1.1399	0.1579	7.58	7.65	3.2713	0.5784
	290	19.74	19.61			8.58	8.75			10.09	10.00		
	370	23.43	23.49			10.13	9.82			12.69	11.82		
	410	25.77	25.66			10.18	10.36			10.73	11.78		
	570	32.53	32.47			13.21	13.20			15.54	15.33		
	920	53.13	53.90			25.51	25.60			27.72	28.21		
	970	60.26	59.55			27.93	27.85			34.52	34.09		
Pingdingshan No. 1 mine	440	19.03	19.03	5.2761	2.2298	11.40	11.79	2.5183	0.5008	12.66	12.68	3.2308	0.9916
	490	18.64	18.65			14.39	13.68			15.12	15.10		
	652	22.39	22.34			17.65	17.64			14.20	14.20		
	692	30.83	30.84			12.85	12.85			14.68	14.68		
	633	22.06	21.93			17.63	17.64			14.99	14.97		
	556	19.74	23.97			16.75	16.18			14.83	16.69		
	555	28.13	24.02			15.26	16.14			19.06	17.21		
Pingdingshan No. 10 mine	1123	65.46	65.46	0.4801	0.2639	31.26	31.12	0.3028	0.0955	38.06	38.06	1.6501	0.7112
	1061	43.06	43.05			22.36	22.55			26.10	26.10		
	785	34.32	34.80			18.29	18.35			22.19	23.54		
	793	36.19	35.71			19.07	19.01			25.07	23.76		
	869	44.40	44.29			25.48	25.49			17.18	17.18		
	514	31.43	31.42			15.44	15.44			17.48	17.44		
	914	40.20	40.32			28.27	28.24			14.24	14.24		
Meishan iron mine	210	9.46	9.70	0.9130	0.1473	4.36	4.38	7.2294	0.9792	3.04	3.04	0.5832	0.0850
	218	11.47	11.22			5.61	5.60			4.80	4.80		
	342	20.19	20.27			7.48	8.59			9.79	9.76		
	350	20.32	20.26			11.79	9.85			9.57	9.72		
	360	16.16	16.17			7.59	8.46			10.28	10.14		
	418	21.50	21.50			11.56	11.56			12.32	12.34		
Beiminghe iron mine	891	21.99	21.99	0.0169	0.0089	10.55	10.56	0.1274	0.0257	9.14	9.14	0.0125	0.0017
	941	23.12	23.12			15.64	15.68			12.45	12.45		
	933	24.72	24.72			15.07	15.03			13.23	13.23		
	984	25.60	25.60			12.32	12.32			14.48	14.49		
	956	31.60	31.58			20.64	20.64			17.92	17.92		

*MV* Measured value, *PV* Predicted value, *MRE* Mean relative error, and *RMSE* Root mean square error.

### Embedded grey neural network combination model

A single prediction model exhibits some limitations to some extent. For the grey model, the larger the data discretization program (i.e., the data gray level), the worse the prediction accuracy. Moreover, the fitting sequence of the model is a non-homogeneous exponential sequence, and it is necessary for the original data to have a clear exponential law to predict the results accurately enough. In addition, the BPNN algorithm is essentially a gradient algorithm for nonlinear optimization problems, which inevitably has local minima and converges slowly in the flat area of the error surface. Furthermore, excessive training times will lead to a decrease in learning efficiency and slow convergence speed of learning algorithms, and the convergence speed is closely related to the selection of initial weights. The combined prediction model can avoid the problem that a single model makes it easy to lose information, reduce randomness, reflect the changing law of the system more comprehensively, and improve prediction accuracy. Based on the idea of combinatorial modeling, a new grey neural network prediction model is established by integrating the GM model with the BPNN model to better solve the model prediction problem.

**Figure 9:**
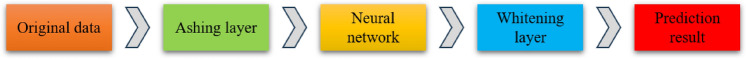
Schematic diagram of the embedded GM–BPNN model.

The sequence generated by the accumulation in the grey prediction method grows monotonically, which weakens the randomness and fluctuation of the original data sequence. At the same time, the Sigmoid activation function commonly used in the BPNN also increases monotonically. Because of the monotonic increasing trend of accumulated data, taking the accumulated data sequence as the training sample of the BPNN, it is convenient for the BPNN to approximate the function, accelerate the convergence speed, and thus improve the prediction accuracy. Accordingly, an embedded grey neural network (GM–BPNN) model (Fig. 9) is adopted in this paper, that is, on the basis of the general BPNN, a grey layer is added to the front end to gray the input data and a whitening layer is added to the back end to restore the output information of the network, so as to get a definite output result.

The grey layer generally accumulates the original data once or multiple times to generate new data, which weakens the randomness of the original data and then uses it as the training sample of the neural network. The whitening layer corresponds to the ashing layer, and the final prediction result can be obtained by performing one or more inverse generation processes.

**Figure 10:**
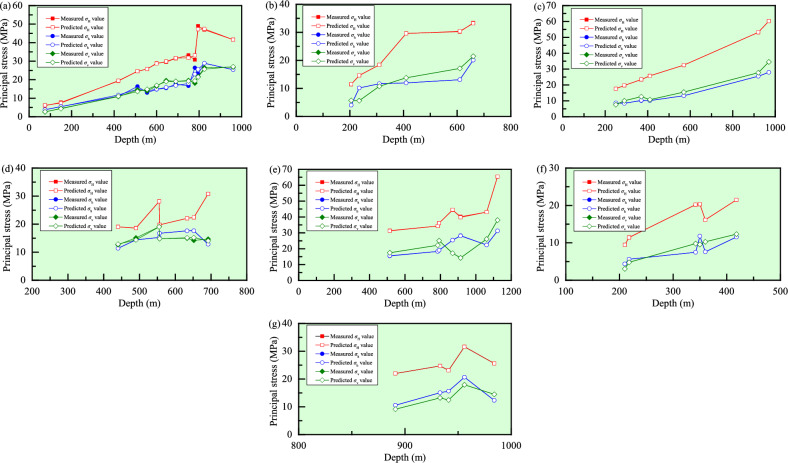
Comparison between the predicted values using the embedded GM–BPNN model and measured values of the three principal stresses in the Sanshandao gold mine (**a**), Xincheng gold mine (**b**), Linglong gold mine (**c**), Pingdingshan No. 1 mine (**d**), Pingdingshan No. 10 mine (**e**), Meishan iron mine (**f**), and Beiminghe iron mine (**g**).

Based on the proposed embedded GM–BPNN, 1-AGO is selected as the grey layer, the BPNN introduced earlier continues to be used, and the final prediction result can be generated by the operation of accumulation and subtraction. The predicted values and average relative errors of three principal stresses in the seven mines are shown in Table 4, and the comparison between the predicted values and measured values is illustrated in Fig. 10. In the seven mines, the mean relative errors of the predicted *σ*_H_ values are 2.0923%, 0.1959%, 0.0174%, 0.1045%, 0.2933%, 0.1239%, and 0.0003%, respectively; the mean relative errors of the predicted *σ*_h_ values are 4.8338%, 0.0477%, 0.9461%, 0.0023%, 0.2707%, 0.0217%, and 0.0001%, respectively; and the mean relative errors of the predicted *σ*_v_ values are 3.2194%, 0.0874%, 0.1986%, 2.7460%, 0.0197%, 0.1034%, and 0.0022%, respectively. The results indicate that the predicted values of this model are very close to the measured values.

**Table 4:** Predicted stress values and mean relative errors based on the embedded GM–BPNN model in the seven mines.

Mine name	Depth (m)	*σ*_H_				*σ*_h_				*σ*_v_			
		MV (MPa)	PV (MPa)	MRE (%)	RMSE (MPa)	MV (MPa)	PV (MPa)	MRE (%)	RMSE (MPa)	MV (MPa)	PV (MPa)	MRE (%)	RMSE (MPa)
Sanshandao gold mine	75	6.01	6.18	2.0923	0.9241	3.81	3.75	4.8338	0.8747	2.56	2.70	3.2194	0.5116
	150	7.73	7.37			5.48	5.43			4.50	4.30		
	420	19.27	19.46			11.05	11.75			10.88	11.07		
	510	24.55	24.56			16.35	14.84			14.49	13.64		
	555	25.71	25.76			13.00	13.95			14.00	14.81		
	600	28.88	28.69			14.77	14.84			16.54	16.90		
	645	29.57	29.83			15.48	15.84			19.56	18.99		
	690	31.50	31.65			17.54	16.84			19.08	19.10		
	750	33.22	31.82			16.68	17.96			19.60	19.25		
	780	30.72	33.10			26.41	23.00			18.09	19.32		
	795	48.93	47.22			23.15	25.52			21.66	21.45		
	825	46.95	47.43			28.88	28.89			26.49	25.69		
	960	41.63	41.62			25.42	25.42			26.79	27.04		
Xincheng gold mine	205	11.45	11.45	0.1959	0.0918	4.03	4.03	0.0477	0.0091	5.69	5.70	0.0874	0.0100
	235	14.62	14.62			10.17	10.17			5.63	5.61		
	310	18.39	18.39			11.65	11.66			10.73	10.74		
	410	29.62	29.57			11.98	11.96			13.77	13.77		
	610	30.21	30.36			13.09	13.09			17.12	17.12		
	660	33.35	33.19			20.08	20.08			21.46	21.46		
Linglong gold mine	250	17.63	17.63	0.0174	0.0065	8.62	8.71	0.9461	0.1297	7.58	7.60	0.1986	0.0280
	290	19.74	19.73			8.58	8.34			10.09	10.04		
	370	23.43	23.44			10.13	10.35			12.69	12.70		
	410	25.77	25.76			10.18	10.12			10.73	10.77		
	570	32.53	32.53			13.21	13.21			15.54	15.51		
	920	53.13	53.13			25.51	25.51			27.72	27.72		
	970	60.26	60.26			27.93	27.93			34.52	34.52		
Pingdingshan No. 1 mine	440	19.03	19.05	0.1045	0.0270	11.40	11.40	0.0023	0	12.66	12.88	2.7460	0.5390
	490	18.64	18.59			14.39	14.39			15.12	14.37		
	652	22.39	22.43			17.65	17.65			14.20	15.20		
	692	30.83	30.82			12.85	12.85			14.68	14.05		
	633	22.06	22.06			17.63	17.63			14.99	15.15		
	556	19.74	19.75			16.75	16.75			14.83	14.84		
	555	28.13	28.11			15.26	15.26			19.06	19.05		
Pingdingshan No. 10 mine	1123	65.46	65.46	0.2933	0.1798	31.26	31.26	0.2707	0.0873	38.06	38.06	0.0197	0.0065
	1061	43.06	43.07			22.36	22.36			26.10	26.10		
	785	34.32	34.32			18.29	18.29			22.19	22.19		
	793	36.19	36.12			19.07	19.07			25.07	25.07		
	869	44.40	44.55			25.48	25.41			17.18	17.19		
	514	31.43	31.28			15.44	15.61			17.48	17.47		
	914	40.20	39.78			28.27	28.13			14.24	14.25		
Meishan iron mine	210	9.46	9.48	0.1239	0.0220	4.36	4.36	0.0217	0.0041	3.04	3.05	0.1034	0.0071
	218	11.47	11.43			5.61	5.61			4.80	4.79		
	342	20.19	20.22			7.48	7.48			9.79	9.80		
	350	20.32	20.32			11.79	11.79			9.57	9.57		
	360	16.16	16.16			7.59	7.59			10.28	10.28		
	418	21.50	21.50			11.56	11.55			12.32	12.32		
Beiminghe iron mine	891	21.99	21.99	0.0003	0	10.55	10.55	0.0001	0	9.14	9.14	0.0022	0
	941	23.12	23.12			15.64	15.64			12.45	12.45		
	933	24.72	24.72			15.07	15.07			13.23	13.23		
	984	25.60	25.60			12.32	12.32			14.48	14.48		
	956	31.60	31.60			20.64	20.64			17.92	17.92		

*MV* Measured value, *PV* Predicted value, *MRE* Mean relative error, and *RMSE* Root mean square error.

**Figure 11:**
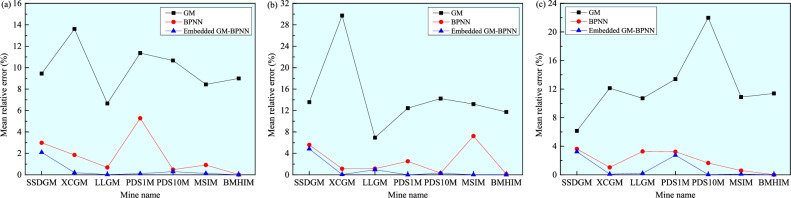
Comparison of the mean relative errors of the *σ*_H_ (**a**), *σ*_h_ (**b**), and *σ*_v_ (**c**) predicted by the GM, BPNN, and embedded GM–BPNN models in the seven mines (SSDGM = Sanshandao gold mine, XCGG = Xincheng gold mine, LLGM = Linglong gold mine, PDS1M = Pingdingshan No. 1 mine, PDS10M = Pingdingshan No. 10 mine, MSIM = Meishan iron mine, and BMHIM = Beiminghe iron mine).

**Figure 12:**
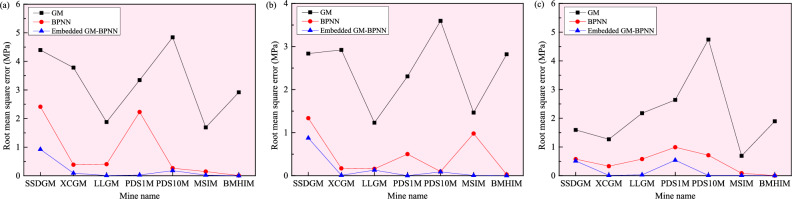
Comparison of root mean square errors of the *σ*_H_ (**a**), *σ*_h_ (**b**), and *σ*_v_ (**c**) predicted by the GM, BPNN, and embedded GM–BPNN models in the seven mines (SSDGM = Sanshandao gold mine, XCGG = Xincheng gold mine, LLGM = Linglong gold mine, PDS1M = Pingdingshan No. 1 mine, PDS10M = Pingdingshan No. 10 mine, MSIM = Meishan iron mine, and BMHIM = Beiminghe iron mine).

In addition, the mean relative error, a statistical indicator used to measure the error between predicted and actual values, is calculated. Its value is between 0 and 1, and the smaller the value, the higher the accuracy of the prediction. The obtained mean relative errors of the *σ*_H_, *σ*_h_, and *σ*_v_ predicted by the GM, BPNN, and embedded GM–BPNN models in the seven mines are plotted in Fig. 11. Apparently, the accuracy of the embedded GM–BPNN model in predicting the principal stress values is much better than that of a single BPNN model and a single GM, with mean relative errors of 0.0003–2.0923%, 0.0001–4.8338%, and 0.0022–3.2194%, respectively. Moreover, another commonly used indicator for evaluating predictive performance, namely, root mean square error, is employed to further measure the root mean square difference between the predicted value and the true value, representing the average degree of deviation between the predicted and true values. The smaller the root mean square error is, the more accurate the prediction result is. The calculated root mean square errors of the *σ*_H_ (a), *σ*_h_ (b), and *σ*_v_ (c) predicted by the GM, BPNN, and embedded GM–BPNN models in the seven mines are drawn in Fig. 12. It can be observed that compared with the other two models, the root mean square errors of the embedded GM–BPNN model in predicting the three principal stresses are the smallest, which are 0–0.9241 MPa, 0–0.8747 MPa, and 0–0.5390 MPa, respectively. The above findings imply that the embedded GM–BPNN model proposed in this study is correct in predicting the in-situ stress magnitudes, and the prediction results have relatively high accuracy, verifying the reliability and applicability of this model.

## Discussion

To significantly improve the design level and economic benefits and ensure the safety of the project, improving the accuracy of in-situ stress measurement is an important issue that must be studied at present. Although there have been dozens of measurement methods and instruments developed based on the principle of OC over the past decades, and some of them have been reported to have achieved complete success in actual measurements, it is impossible to compare the measured values with unknown quantities to determine the accuracy of the measurement results because the actual stress values are unknown^16,36,52^. There is no other choice but to evaluate the reliability and accuracy of the measurement results based solely on personal experience and subjective judgment. In fact, there are few objective evaluations of the working performance and measurement accuracy of various methods and instruments under actual rock conditions before. This evaluation can only be completed through laboratory experiments under the condition that the stress value is known. To achieve this, a series of OC tests can be conducted in the laboratory by simulating the actual in-situ stress and rock conditions. By comparing the stress value calculated based on the measured strain or deformation with the actually applied stress value, the working performance and measurement accuracy of the OC instrument under this test condition can be quantitatively analyzed and evaluated.

Accurate in-situ stress measurement results are the prerequisite for establishing an accurate in-situ stress prediction model. As mentioned earlier, in response to the problems existing in the conventional OC strain gauges, our team has proposed the concept of accurate in-situ stress measurement, made technical improvements in the measurement circuit and temperature compensation, and invented an improved complete temperature compensation hollow inclusion strain gauge, which greatly reduced the influence of temperature change on the measurement accuracy. Moreover, Li et al.^53^ conducted a comprehensive comparison and analysis of the magnitude and causes of errors in measurement circuits and temperature compensation between two types of hollow inclusion strain gauge probes currently used in China and pointed out that the 13-wire probe produces smaller errors in the circuit, while the 15-wire probe performs better in temperature compensation, and they provided a complete form of the correction formula for confining pressure calibration. The in-situ digital hollow inclusion strain gauge produced by Environmental Systems & Services Pty Ltd in Australia uses an in-situ AD conversion to eliminate the influence of attenuation in signal transmission^54^, but the data line needs to pass through the drill pipe water hole one by one, which interferes with the drilling work during measurement. Additionally, Bai et al.^55^ developed a deep borehole hollow inclusion strain gauge in-situ stress measuring instrument, which installs a cable-free micro-probe at a predetermined position inside the hole for measurement. However, the conventional temperature compensation method can not reduce the measurement error caused by large temperature changes in deep strata. With the development of deep exploration and excavation in the earth, the temperature disturbance during OC and the nonlinear behavior of rocks under high-stress levels increase the error of in-situ stress measurement, which further aggravates the demand for the high accuracy of in-situ stress measurement^56^. Consequently, the OC technique with hollow inclusion strain gauge needs to be designed from the aspects of strain gauge structure, strain gauge arrangement, the circuit board size, circuit wiring, and component selection and layout to improve the performance of the strain gauge in measurement accuracy, stability, and long-term, develop a new data acquisition system, and thus realize the front-end digital data acquisition of the hollow inclusion strain gauge.

**Figure 13:**
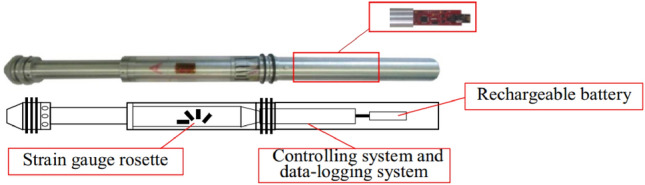
Structure of the new improved front-end digital hollow inclusion strain gauge^45^.

To meet the requirements of accurate in-situ stress measurement, based on the OC technique with hollow inclusion strain gauge, Li et al.^45^ systematically studied the stability of the acquisition circuit, digitalization of complete temperature compensation technique, and adaptability of measuring equipment in a complex environment, and developed a front-end digital acquisition system (Fig. 13) with instantaneous acquisition and power interruption continuous acquisition functions. Moreover, based on the temperature self-compensation technique and double temperature compensation design, the application of the complete temperature compensation technique in front-end digital hollow inclusion strain gauge in-situ stress measurement has been achieved. The double temperature compensation algorithm combined with temperature calibration experiments realizes double temperature compensation for both the measurement circuit and the acquisition circuit. The temperature deviation in the sensor system and the data recording system was calculated by the double temperature compensation method. The indoor calibration experiments and field test data indicated that the correction of the original temperature compensation algorithm data by the double temperature compensation technique reached 15%. In addition, they used the confining pressure calibration method and a true three-dimensional in-situ stress test bench to conduct indoor tests to simulate field conditions, and the results showed that the data truly reflected the spatial stress changes. The digital hollow inclusion strain gauge OC technique with double temperature compensation algorithm exhibits good test accuracy and stability of acquisition system in both indoor and field tests, which represents the development direction of in-situ stress measurement technique in the future.

In addition, GM, BPNN, and embedded GM–BPNN models are constructed separately to predict the magnitude of in-situ stress. According to the results obtained in the study, compared with the GM and BPNN models, the embedded GM–BPNN model produces the best results, that is, less sample size requirements and the best performance index (Table 4). When the embedded GM–BPNN model is used, more than 90% of the prediction results are considered to be close to the truth. The embedded GM–BPNN model significantly improves the accuracy of the GM(0, 1) model by correcting the error of the predicted value, and has the characteristics of high calculation accuracy and fast operation speed.

There are some similarities between grey theory and BPNN in information representation. The output of BPNN can approach a fixed value with a certain precision, but because of the error, the output will fluctuate around a certain value. According to the definition of grey number in grey theory, it can be considered that the output of BPNN is actually a grey number^57^. Thus, the BPNN itself contains the content of grey theory. Furthermore, there are also some differences and complementarities between them. The BPNN model can approximate a nonlinear function with arbitrary accuracy, while the GM is not suitable for approximating complex nonlinear functions, but it can better predict the overall trend of parameter changes. The cumulative generation in the GM is that the sequence shows a monotonic growth trend, which is more suitable for the BPNN model approximation. As a consequence, by combining the GM and BPNN models and learning from each other’s strong points, the predictive performance of the embedded GM–BPNN model is satisfactory. This has also been confirmed in this study.

It should be noted that the modeling mechanism of each prediction method is different. If a prediction model is considered completely unsuitable for prediction because of its large prediction error, some useful information may be lost. In fact, even a prediction model with a large prediction error, if it contains some independent information about the system when it is combined with a prediction model with a smaller prediction error, it is entirely possible to increase the prediction accuracy of the system. In this study, although the prediction error of the GM is relatively large, its small sample requirement and ability to process poor information make the GM have a broad application prospect, especially for the case of less in-situ stress measurement data. Consequently, in addition to combining GM with the BPNN model, it is also possible to try combining GM with other prediction techniques, such as support vector machines, prior knowledge, evidence theory, and chaos theory, which helps promote the development of the grey theory. This is also the research work that we will carry out in the near future. In addition, the embedded GM–BPNN model proposed in this study is only used to predict the OC stress data measured by our team. To further validate the wide applicability and practicability of this model, the in-situ stress data obtained by other measurement techniques (such as hydraulic fracturing, anelastic strain recovery, and acoustic emission) will be predicted and analyzed in the future. Based on this, the model will be continuously revised and optimized to improve the prediction accuracy.

### Consent to participate

All authors have confirmed their participation.

## Summary and conclusions

1. The concept of accurate in-situ stress measurement is proposed, the measurement circuit, temperature compensation, and calculation method are improved, and an improved complete temperature compensation hollow inclusion strain gauge is invented, which reduces the influence of temperature change on measurement accuracy. The adoption of the complete temperature compensation technology and the reasonable determination of rock elastic parameters that are suitable for the measured stress level based on the nonlinearity of the rock have significantly improved the accuracy of stress measurement. This measurement technique has been applied to in-situ stress measurements in many mines, and a large amount of valuable data has been determined. The measurement results indicate that the horizontal tectonic stress plays a dominant role in the stress field of those mining areas, the vertical principal stress magnitude is basically equal to or slightly less than the weight of overlying strata, and the magnitude of the maximum horizontal principal stress is around twice that of the vertical principal stress on average, and the three principal stresses increase approximately linearly with depth. The dominant direction of the maximum principal stress identified in each mine is in good agreement with the directions of various stress indicators previously derived from other different measurement methods in and around the mining area.
2. The mean relative errors of the prediction results of GM(0, 1) for the three principal stresses all reach 6–30%, and the accuracy of the model fails to meet the requirements. The average relative errors of the prediction results of the BPNN model are all less than 10% (0.0125–7.2294%), and the model accuracy meets the requirements and has sufficient credibility. Compared with the GM and BPNN models, the embedded GM–BPNN model produces the best prediction results. The accuracy of the embedded GM–BPNN model in predicting the principal stress values is much better than that of a single BPNN model and a single GM, with mean relative errors of 0.0003–2.0923%, 0.0001–4.8338%, and 0.0022–3.2194%, respectively. Moreover, compared with the other two models, the root mean square errors of the embedded GM–BPNN model in predicting the three principal stresses are the smallest, which are 0–0.9241 MPa, 0–0.8747 MPa, and 0–0.5390 MPa, respectively. Hence, the predicted values of the embedded GM–BPNN model are very close to the measured values, verifying the reliability and applicability of this model.
3. The embedded GM–BPNN model for predicting in-situ stress values established fully utilizes the characteristics of grey theory and BP neural network, which require a small sample size, weaken the randomness of the original data, and gradually approach the accuracy of the model, making it particularly suitable for situations with limited stress measurement data. To further validate the wide applicability and practicability of this model, future prediction and analysis of the stress data obtained by other measurement techniques (such as hydraulic fracturing, anelastic strain recovery, and acoustic emission) will be carried out, which will be beneficial for further revising and optimizing the model to improve the prediction accuracy. In addition, only the magnitude of stress is predicted and analyzed in this study, and another important component of stress tensor, i.e., stress direction, will be predicted in the future, striving to achieve complete stress tensor prediction. With the increase of stress data volume and the complexity of stress data in the future, the accuracy and calculation speed of the embedded GM–BPNN model to predict different types of stress data need to be further improved to obtain higher reliability and credibility of prediction results.

## Data Availability

The datasets used during the current study are available from the corresponding author upon reasonable request.
